# Methodology in conducting a systematic review of systematic reviews of healthcare interventions

**DOI:** 10.1186/1471-2288-11-15

**Published:** 2011-02-03

**Authors:** Valerie Smith, Declan Devane, Cecily M Begley, Mike Clarke

**Affiliations:** 1 School of Nursing and Midwifery, University of Dublin, Trinity College Dublin, 24 D'Olier Street, Dublin 2, Ireland; 2 School of Nursing and Midwifery, National University of Ireland, Galway, Galway, Ireland; 3 UK Cochrane Centre, National Institute for Health Research, Middle Way, Oxford, OX2 7LG, UK

## Abstract

**Background:**

Hundreds of studies of maternity care interventions have been published, too many for most people involved in providing maternity care to identify and consider when making decisions. It became apparent that systematic reviews of individual studies were required to appraise, summarise and bring together existing studies in a single place. However, decision makers are increasingly faced by a plethora of such reviews and these are likely to be of variable quality and scope, with more than one review of important topics. Systematic reviews (or overviews) of reviews are a logical and appropriate next step, allowing the findings of separate reviews to be compared and contrasted, providing clinical decision makers with the evidence they need.

**Methods:**

The methods used to identify and appraise published and unpublished reviews systematically, drawing on our experiences and good practice in the conduct and reporting of systematic reviews are described. The process of identifying and appraising all published reviews allows researchers to describe the quality of this evidence base, summarise and compare the review's conclusions and discuss the strength of these conclusions.

**Results:**

Methodological challenges and possible solutions are described within the context of (i) sources, (ii) study selection, (iii) quality assessment (i.e. the extent of searching undertaken for the reviews, description of study selection and inclusion criteria, comparability of included studies, assessment of publication bias and assessment of heterogeneity), (iv) presentation of results, and (v) implications for practice and research.

**Conclusion:**

Conducting a systematic review of reviews highlights the usefulness of bringing together a summary of reviews in one place, where there is more than one review on an important topic. The methods described here should help clinicians to review and appraise published reviews systematically, and aid evidence-based clinical decision-making.

## Background

The healthcare literature contains hundreds of thousands of studies of healthcare interventions, growing at tens of thousands per year [1]. In most areas of health care, there are too many studies for people involved in providing care to identify and consider when making decisions. Researchers have recognised this problem and many have accepted the challenge of preparing systematic reviews of individual studies in order to appraise, summarise and bring together existing studies in a single place. More recently, calls have been made for 'rapid reviews' to provide decision-makers with the evidence they need in a shorter time frame, but the possible limitations of such 'rapid reviews', compared to full systematic reviews, require further research [2]. There are now several organisations dedicated to the preparation of systematic reviews, including the National Institute of Health and Clinical Excellence (NICE) in the UK, the Evidence-based Practice Centre Program, funded by AHRQ in the USA, the Joanna Briggs Institute, and the international Campbell and Cochrane Collaborations, with the latter being the largest single producer of systematic reviews in health care, with more than 4200 published by the end of 2010 [3]. In recent years however, decision makers who were once overwhelmed by the number of individual studies have become faced by a plethora of reviews [4,5]. These reviews are likely to be of variable quality and scope, with more than one systematic review on important topics. For example, a comprehensive search of twelve health related citation databases (using database specific search strategies) identified over thirty reviews evaluating the effectiveness of nurse and midwife-led interventions on clinical outcomes, as part of an on-going study into the impact of the role of nurse and midwife specialist and advanced practitioners in Ireland. A logical and appropriate next step is to conduct a systematic review of reviews of the topic under consideration, allowing the findings of separate reviews to be compared and contrasted, thereby providing clinical decision makers with the evidence they need. We have been involved in several examples of systematic reviews (or overviews) of reviews [6-9] and The Cochrane Collaboration introduced a new type of Cochrane review in 2009 [10], the overview of Cochrane reviews, with two full overviews [11,12] and protocols for five more [13-17] published by October 2010. These reviews of reviews aims to provide a summary of evidence from more than one systematic review at a variety of different levels, including the combination of different interventions, different outcomes, different conditions, problems or populations, or the provision of a summary of evidence on the adverse effects of an intervention [10].

This paper describes the conduct and methods used to identify and appraise published and unpublished systematic reviews systematically. It draws on our experience of conducting several of these reviews of reviews in recent years. The purpose of such an overview, in identifying and appraising all published reviews is to describe their quality, summarise and compare their conclusions and discuss the strength of these conclusions, so that best evidence is made available to clinical decision-makers. During the review process a number of methodological challenges can arise. We describe these challenges and offer possible solutions to overcome them. We hope to provide a guide to clinicians and researchers who wish to conduct a systematic review of reviews and to share our experiences.

## Methods

The objective and the reasons for conducting a systematic review of reviews should be made explicit at the start of the process, as this is likely to influence the methods used for the review. In formulating the scope for the review of reviews, the PICOS (participants, interventions, comparators, outcomes, and study design) structure may be helpful. This can help the reviewers to delineate clearly if they wish, for example, to compare and summarise systematic reviews that address the same treatment comparison or a particular intervention for a population or condition, or a range of interventions for people with a specific condition. Following this, the methods in conducting a systematic review of reviews require consideration of the following aspects, akin to the planning for a systematic review of individual studies: sources, review selection, quality assessment of reviews, presentation of results and implications for practice and research.

### Sources and searching

Locating and retrieving relevant literature is challenging, yet crucial to the success of a systematic review. The material sourced provides the information from which evidence, conclusions and recommendations are drawn. For many, the literature search may appear overwhelming, given the sheer volume of material to check through. However, establishing a systematic search strategy, before commencing the literature search, is fundamental to appropriate and successful information retrieval. This planning assists in meeting the requirements of the systematic review and in answering the research question. In developing a search strategy, the scope of the search, its thoroughness and the time available to conduct it, all need to be considered. The aim is to ensure that the systematic review of reviews is comprehensive, thorough and objective.

The methods used in sourcing relevant literature to conduct a systematic review of reviews are similar to those adopted in conducting a systematic review of individual studies with some subtle differences described here. A realistic time-frame to conduct the systematic review of reviews should be established. It has been estimated that a typical systematic review would take between six and eighteen months [18] but this is very dependent on the research question and the staffing, funding and other resources available. The process might be faster for a systematic review of reviews if the time-frame to complete the literature search is significantly reduced through the ability to target the searching of articles most likely to be reports of a systematic review. In a systematic review of individual studies, the search should be as wide as possible to maximize the likelihood of capturing all relevant data and minimizing the effects of reporting biases. A search of a variety of electronic databases relevant to the topic of interest is recommended [18]. However, in a systematic review of reviews, it may be possible to limit the searches to databases specific to systematic reviews such as the Cochrane Database of Systematic Reviews and the Database of Abstracts of Reviews of Effects. Likewise, although the search for a review of individual studies might need to cover many decades [19], limiting the search to period from the early 1990 s onwards is likely to identify all but the very small minority of systematic reviews conducted before then [20,21]. Furthermore, researchers might find that identifying and highlighting a recent high quality systematic review will prove of most benefit to decision makers using their review or reviews. However, a summary of the earlier reviews can still prove helpful if these contain relevant information that is not included in the recent review. Applying language restrictions is not recommended; but, unavoidable constraints such as a lack of access to translation services or funds to pay for these may make it necessary to restrict the systematic review or reviews to English language publications. In such instances, this limitation should be acknowledged when reporting the review and it might be worthwhile reporting the difference between searches with and without language restrictions in order to estimate the amount of literature that might have been excluded.

The search terms used for the literature search should be clearly described, with information on their relevance to the research question. Furthermore, search terms should be focused so that they are broad enough in scope to capture all the relevant data yet narrow enough to minimize the capture of extraneous literature that may result in unnecessary time and effort being spent assessing irrelevant articles. In conducting a systematic review of reviews, systematic reviews rather than individual studies are of interest to the reviewer and several search strategies have been developed to identify this type of research [22,23] which could be combined with the terms for the relevant healthcare topic. In developing the search strategy for a systematic review of reviews, researchers might wish to consider the PRESS initiative, developed as a means for peer reviewing literature searches [24] to check that the various elements of the electronic search strategy have been considered. To minimize the risk of missing relevant reviews, a manual search of key journals and of the reference lists of reviews captured by the initial searches is also recommended. The literature search can also be complemented by contacting experts in the topic under review and by checking articles which cite individual studies that are known to be relevant to the topic. This may prove relevant in learning of published systematic reviews that are not indexed in the bibliographic databases searched, and of ongoing systematic reviews near completion. The development of a prospective register of systematic reviews should help further with this [25].

### Review Selection

A major challenge to review selection is identifying all reviews relevant to the topic of interest, and of potential importance to answering the research question. During the planning phase, before commencing the systematic review of reviews, a review team should be established. The review team should include at least one person with methodological expertise in conducting systematic reviews and at least one person with expertise on the topic under review. The review team is responsible for developing a review selection strategy. An agreement of inclusion and exclusion criteria should be made before starting the review selection process. Aspects of this process might include decisions regarding the type of reviews that may be included in the systematic review. For example, in our review on interventions for preventing preterm birth [6], we restricted the inclusion criteria to reviews of randomized controlled trials. Another example of inclusion criteria might be to limit the systematic review of reviews to reviews of a particular type of participant (such as women having their first baby) or which assess a particular type of pain relief.

When a selection strategy has been developed, the selection process is carried out in a similar way to a review of individual studies:

• Assess retrieved titles and abstracts for relevance and duplication.

• Select those you wish to retrieve and appraise further.

• Obtain full text copies of these potentially eligible reviews.

• Assess these reviews for relevance and quality; ideally, using independent assessment by at least two members of the review team. This reduces bias in review selection and allows for appropriate discussion should uncertainty arise.

### Quality Assessment of Reviews

The quality and strength of evidence presented in the individual, included reviews should influence the conclusions drawn in the systematic review of these. The quality and scope of published reviews varies widely. The strength of the conclusions and the ability to provide decision-makers with reliable information depends on the inclusion of reviews that meet a minimum standard of quality. When assessing the quality of the reviews, one should try to avoid being influenced by extraneous variables, such as authors, institutional affiliations and journal names; and should focus on the quality of the conduct of the review. Although the researchers will usually have to do this via an assessment of the quality of report, with the hope that initiatives such as PRISMA (formerly, QUOROM) which assist by facilitating adequate standards of reporting [26].

The AMSTAR tool [27], which became available after we started work on our review of reviews, is the only tool that we are aware of that has been validated as a means to assess the methodological quality of systematic reviews and could be used in the review of reviews to determine if the potentially eligible reviews meet minimum requirements based on quality. While the authors of the AMSTAR paper [27] recognise the need for further testing of the AMSTAR tool, important domains identified within the tool are: establishing the research question and inclusion criteria before the conduct of the review, data extraction by at least two independent data extractors, comprehensive literature review with searching of at least two databases, key word identification, expert consultation and limits applied, detailed list of included/excluded studies and study characteristics, quality assessment of included studies and consideration of quality assessments in analysis and conclusions, appropriate assessment of homogeneity, assessment of publication bias and a statement of any conflict of interest.

Although our review of reviews began before the publication of the AMSTAR tool, we used similar domains to assess review quality. Our assessment criteria are shown below and provide a structure that can be used to report the quality and comparability of the included reviews to help readers assess the strength of the evidence in the review of reviews:

▪ The extent of searching undertaken: Are the databases searched, years searched and restrictions applied in the original review clearly described? Information on the extent of searching should be clearly provided, to allow for a comprehensive assessment of the scope of the review.

▪ Description of review selection and inclusion criteria: Do the authors of the original review provide details of study selection and eligibility criteria and what are these details? This information should be clearly reported in the systematic review of reviews.

▪ Assessment of publication bias: Did the authors of the original review seek additional information from authors of the studies they included? Are there any details of statistical tests (such as funnel plot analysis) to assess for publication bias?

▪ Assessment of heterogeneity: Did the authors of the original review discuss or provide details of any tests of heterogeneity? In the presence of significant heterogeneity, were statistical tests used to address this?

▪ Comparability of included reviews: Are the reviews comparable in terms of eligibility criteria, study characteristics and primary outcome of interest? For example, in our review of reviews on fetal fibronectin and transvaginal cervical ultrasound for predicting preterm birth, [8] we included reviews that had incorporated studies among women who were both symptomatic and asymptomatic for preterm birth. As a means of addressing comparability of the included reviews, we provided details of the number of women in each group separately and reported the results for each group separately, where applicable.

### Presentation of Results

When the results of a systematic review of reviews are presented, this should present the reader with the major conclusions of the review through the provision of answers to the research question, as well as the evidence on which these conclusions are based and an assessment of the quality of the evidence supporting each conclusion; for example, using the GRADE approach as adopted for the 'Summary of Findings' table in Cochrane reviews [28]. It is important to be specific in reporting the primary outcome of interest for the review, and this can reduce workload by limiting data extraction to only those results relevant to the topic of interest from reviews that report on several outcome measures. For example, some systematic reviews on antibiotic therapy for the prevention of preterm birth [29,30] report a variety of outcome measures other than preterm birth (e.g. neonatal outcomes). However, in our systematic reviews of reviews [6,8], our research focus on preterm birth meant that only results for the effects on preterm birth were extracted.

The use of summary tables and figures is helpful in presenting results in a structured and clear format that will enhance textual commentary. Table 1 is an example of the provision of details of the scope of the reviews included in a systematic review of reviews (3). Sources of evidence and some quality assessment criteria are included. The quality assessment is enhanced by a narrative discussion of heterogeneity and publication bias.

**Table 1:** Summary table of scope of reviews in a systematic review of reviews^1^

Review Year	Aim (participants)	Search strategy	No. of studies included	Total no. of participants	Timing of preventative strategy
King & Flenady 2002	To assess the effects of prophylactic antibiotics on preterm labour (women symptomatic for preterm labour)	Cochrane Pregnancy & Childbirth Group (May 2002) Search terms provided. No language restrictions	11	7428 (6295 enrolled in one trial)	Mean gestational age at entry to all trials 30-32 weeks (but varied across studies)

Simcox et al 2007	To determine if antibiotics reduce the risk of preterm birth (asymptomatic women at risk, e.g. previous preterm birth or positive fibronectin status)	Cochrane Pregnancy & Childbirth Group (2005) Search terms provided. English language publications.	17	1291	12-28 weeks across studies

^1^Reprinted from the European Journal of Obstetrics & Gynecology and Reproductive Biology, Volume 142, Smith V, Devane D, Begley CM, Clarke M, Higgins S. A systematic review and quality assessment of systematic reviews of randomised trials of interventions for preventing and treating preterm birth. 3-11, Copyright (2009), with permission from Elsevier.

Table 2 provides an example of how summary results from each original review might be presented in the systematic review of reviews.

**Table 2:** Summary of results reported in a systematic review of reviews^2^

Review	Tocolytic agent	Birth >48 hrs (95% CI)	Birth >7 days (95% CI)	Birth >34 weeks (95% CI)	Birth >37 weeks (95% CI)
King 1988	Betamimetics compared with placebo or no treatment	12 trials OR 0.59, (0.42-0.83) Significant	-	-	8 trials OR 0.71 (0.53-0.96) Significant

Coomarasamy et al 2002	Atosiban V placebo (2 trials) Atosiban V beta-agonist (4 trials)	2 trials RR 1.13, (1.02-1.26) Significant 4 trials RR 1.07 (0.98-1.17) Not significant	- 3 trials RR 1.25 (1.09-1.44) Significant	-	-

Crowther et al 2002	Magnesium sulphate V placebo/no treatment or other tocolytic agent	11 trials RR 0.85, (0.58-1.25) Not significant	-	No difference reported	No difference reported

King et al 2003	Calcium channel blockers V any other tocolytic agent	-	RR 0.76, (0.60-0.97) Significant	RR 0.83, (0.69-0.99) Significant	RR 0.95, (0.83-1.09) Not significant

Anotayanonth et al 2004	Betamimetics V Placebo	11 trials RR 0.63, (0.53-0.75) Significant	11 trials RR 0.78, (0.68-0.90) Significant	-	11 trials RR 0.95, (0.88-1.03) Not significant

King et al 2005	COX inhibitor V Placebo COX inhibitor V any other tocolytic	2 trials RR 0.20, CI 0.03-1.28 4 trials RR 0.59, CI 0.34-1.02	2 trials RR 0.41, CI 0.10-1.66	-	3 trials RR 0.21, CI 0.07-0.62 3 trials RR 0.53, CI 0.31-0.94

Whitworth & Quenby 2008	Oral betamimetic V placebo	-	-	-	RR 1.07, (0.14-8.09) Not significant

^2 ^Reprinted from the European Journal of Obstetrics & Gynecology and Reproductive Biology, Volume 142, Smith V, Devane D, Begley CM, Clarke M, Higgins S. A systematic review and quality assessment of systematic reviews of randomised trials of interventions for preventing and treating preterm birth. 3-11, Copyright (2009), with permission from Elsevier.

The use of a checklist or reporting tool may also guide the reviewer when reporting on a systematic review of reviews. Although we did not identify a tool specific to reporting of systematic reviews of reviews, the PRISMA statement provides a useful framework to follow [26]. This guidance, developed for reporting systematic reviews and meta-analyses of studies that evaluate healthcare interventions, can be used to assess item inclusion in a systematic review of systematic reviews.

### Implications for practice and research

One of the problems faced by decision makers who encounter multiple reviews of the same topic is inconsistency in the results or conclusions of these reviews. Jadad et al (1997) provide guidance on how to address discordant results from individual reviews [31] and conducting systematic reviews of reviews will help to address this issue further. A systematic review of reviews can provide reassurances that the conclusions of individual reviews are consistent, or not. The quality of individual reviews may be assessed, so that evidence from the best quality reviews can be highlighted and brought together in a single document, providing definitive summaries that could be used to inform clinical practice.

## Discussion

### Meta-analyses in systematic reviews of reviews

A major challenge in conducting a systematic review of reviews is the creation of a 'meta-analysis' of the included reviews, which are themselves meta-analyses. In doing this, it is important that data from individual studies are not used more than once. This would give too much statistical power, with the risk that a misleading estimate will be produced and that this will be overly precise. Overcoming this challenge would require the unpicking of each of the included reviews and the subsequent combination of the results of the individual, included studies. This may prove to be a complex and time-consuming task and careful consideration should be given to its value when planning the systematic review of reviews, highlighting the importance of having clear reasons for conducting the review.

## Conclusion

A systematic review of reviews allows the creation of a summary of reviews in a single document. In this paper, we have discussed the methods for conducting such a review. The methods we have described and discussed draw on our experiences, and should be useful to healthcare practitioners who wish to conduct a systematic review of reviews to enhance their evidence-based knowledge and to support well-informed clinical decision making. They should also be useful to practitioners who will find that the ideal starting point for knowledge from research will be a systematic review of reviews of the topic of interest to them.

## Competing interests

The authors declare that they have no competing interests.

## Authors' contributions

VS participated in the sequence content and drafted the manuscript. MC conceived and contributed to the rationale for the manuscript. VS, CB, DD and MC contributed to the design of the manuscript. CB, DD and MC read and critically revised the draft manuscript for important intellectual content. All authors read and approved the final manuscript.

## Pre-publication history

The pre-publication history for this paper can be accessed here:

http://www.biomedcentral.com/1471-2288/11/15/prepub
